# Suppression of the long non-coding RNA *LINC01279* triggers autophagy and apoptosis in lung cancer by regulating FAK and SIN3A

**DOI:** 10.1007/s12672-023-00855-4

**Published:** 2024-01-02

**Authors:** Jiancong Wu, Xiaobi Huang, Xiaofang Li, Honglian Zhou, Xiaorao Chen, Yongyang Chen, Yudong Guo, Jian Huang, Hanqing Huang, Zhong Huang, Guoan Chen, Zhixiong Yang, Jian Zhang, Wenmei Su

**Affiliations:** 1 Department of Pulmonary Oncology, Affiliated Hospital of Guangdong Medical University, Zhanjiang, China; 2 Center for Pathological Diagnosis and Research, Affiliated Hospital of Guangdong Medical University, Zhanjiang, China; 3 Department of Thoracic Surgery, Maoming People’s Hospital, Maoming, China; 4 School of Medicine, Southern University of Science and Technology, Shenzhen, China; 5 Guangdong Provincial Key Laboratory of Autophagy and Major Chronic Non-Communicable Diseases, Affiliated Hospital of Guangdong Medical University, Zhanjiang, China

**Keywords:** lncRNA, *LINC01279*, FAK, SIN3A, Apoptosis, Autophagy, Lung adenocarcinoma

## Abstract

Long non-coding RNAs play critical roles in the development of lung cancer by functioning as tumor suppressors or oncogenes. Changes in the expression of *LINC01279* have been associated with cell differentiation and human diseases. However, the mechanism underlying *LINC01279* activity in tumorigenesis is not clear. Here, we analyzed the function of *LINC01279* in lung adenocarcinoma using clinical samples, xenografts, and non-small-cell lung cancer cell lines. We found that *LINC01279* is highly expressed in lung adenocarcinoma and may be considered as a predictive factor for this cancer. Knockdown of *LINC01279* prevents tumor growth in xenografts and in cancer cell lines by activating autophagy and apoptosis. Molecularly, we revealed that *LINC01279* regulates the expression of focal adhesion kinase and extracellular-regulated kinase signaling. In addition, it complexes with and stabilizes the transcriptional co-repressor SIN3A protein. Suppression of focal adhesion kinase and SIN3A also induces apoptosis and prevents tumor progression, suggesting that they may at least in part mediate the oncogenic activity of *LINC01279*. These results identify *LINC01279* as a possible oncogene that plays an important role in the development of lung cancer. Our findings provide insights into the mechanism underlying *LINC01279*-mediated oncogenesis of lung adenocarcinoma. They may help to discover potential therapeutic targets for cancer diagnosis and prognosis.

**Supplementary Information:**

The online version contains supplementary material available at 10.1007/s12672-023-00855-4.

## Introduction

Lung cancer is the leading cause of cancer-related deaths, resulting in 1.8 million deaths in 2020 worldwide [1]. The overall survival (OS) time of patients with driver gene mutations and advanced lung cancer is only 10–12 months in the absence of specific medical intervention. With targeted therapies using drugs that modulate the activity of genes or proteins involved in cancer cell growth and survival, the median OS can be extended to more than 3 years. Lung adenocarcinoma (LUAD) is a type of non-small-cell lung cancer (NSCLC) and accounts for about 40% of lung cancers [2]. It is generally considered that LUAD shows distinct genomic alterations with respect to other NSCLC subtypes [2, 3]. Prolonging the survival of patients and improving their quality of life via targeted therapies are undoubtedly beneficial in the treatment of lung cancer. Thus it is critical to understand the molecular mechanism regulating its occurrence and progression for the identification of novel therapeutic targets.

There is increasing evidence suggesting a key role of post-transcriptional control mediated by non-coding RNAs in the pathogenesis and development of various cancers [4]. Long non-coding RNAs (lncRNAs) are critically involved in the regulation of gene expression in lung cancers by acting as both tumor suppressors and oncogenes [5]. Thus, they are promising biomarkers and therapeutic targets for lung diseases [6]. The IncRNA *LINC01279* was originally identified as a down-regulated non-coding gene during osteogenic differentiation [7]. Subsequent works suggested that its function could be associated with the pathogenesis of endometriosis [8, 9]. Interestingly, more recent studies indicated a correlation between *LINC01279* expression and progression of gastric cancer [10], suggesting a possible function of this IncRNA in regulating tumor growth. However, whether it plays a role in other cancers is unknown. In particular, the molecular and cellular mechanisms by which it functions to regulate tumorigenesis remain unclear.

In this study, we report the function of *LINC01279* in the progression of LUAD. Using clinical samples, xenografts and NSCLC cell lines, we show that the expression of *LINC01279* is significantly up-regulated in LUAD tumor tissues and NSCLC cell lines. Functional analyses indicate that suppression of *LINC01279* decreases the expression of focal adhesion kinase (FAK) and extracellular-regulated kinase (ERK) signaling. Moreover, we demonstrate that *LINC01279* complexes with and stabilizes the transcriptional co-repressor SIN3A. Knockdown of *LINC01279* or SIN3A activates autophagy and apoptosis in NSCLC cells. Importantly, inhibition of *LINC01279* function reduces tumor growth in xenografts derived from NSCLC cells. These observations suggest that *LINC01279* displays pro-tumor function and plays an important role in the development of lung cancer. Its expression may be considered as a predictive factor of LUAD. Our findings provide insights into the mechanism underlying *LINC01279*-mediated lung tumorigenesis. They may also help to identify potential therapeutic targets for cancer diagnosis and prognosis.

## Materials and methods

### Ethical statement

This project was approved by the Research Ethics Committee of the Affiliated Hospital of Guangdong Medical University. All experiments using animals were performed in accordance with the ARRIVE guidelines. Clinical tissues were collected with written informed consents of each patient.

### Collection of clinical tissues

Lung carcinoma tissues and adjacent noncancerous tissues were collected from 90 consecutive patients with LUAD. These patients were subjected to curative resection between September 2014 and September 2016 at the Department of Thoracic Surgery of the Affiliated Hospital of Guangdong Medical University (Zhanjiang, China). Tissue blocks were selected from lung cancer tissue specimens and analyzed with respect to the clinicopathological and follow-up data of patients. The histopathological diagnosis was based on the standard of the World Health Organization.

### Cell lines and transfection

H1299, H1650, H838 and PC-9 cells (human NSCLC cell lines with different metastatic potentials) were purchased from Kobio Biology (Nanjing, China) and tested routinely for mycoplasma contamination as described previously [11]. They were cultured in RPMI-1640 medium (Gibco) supplemented with 10% fetal bovine serum (Invitrogen) at 37 °C with 5% CO_2_ in a humidified atmosphere. Cell transfection was performed using the Lipofectamine^®^ RNAiMAX Reagent (Invitrogen) according to the manufacture’s instructions.

For OS analysis, HLugA180Su06 LUAD tissue microarray with clinical pathological data and survival information (Shanghai Outdo Biotech Biotechnology, China) were used to examine *LINC01279* expression levels. This microarray contains 94 tumors and 86 paired adjacent normal tissues, which were collected from patients subjected to surgical resection from July 2004 to June 2009. The follow-up of these patients lasted 5 to 10 years until August 2014.

### Knockdown by siRNA and shRNA

For siRNA-mediated knockdown, cells were plated at a desired density and further cultured for 12 h to 24 h. They were then transfected with gene-specific siRNA or a non-targeting siRNA (Supplementary Table S1) at a final concentration of 10 μM, using the Lipofectamine® RNAiMax Reagent (Invitrogen) in OptiMEM medium according to the manufacturer’s instructions. For shRNA-mediated knockdown, lentiviral LV10*-*U6*/*RFP&Puro shuttle vector (GenePharma, Shanghai, China) was used to allow the expression of *LINC01279*-specific shRNAs or a non-targeting shRNA (Supplementary Table S1). Cells were cultured and transfected as above.

### Tumor xenografts

Five-week-old male BALB/C nude mice were maintained under specific pathogen-free conditions. Stably transfected H1299 cells (5 × 10^6^ cells in 200 µL of PBS) were implanted into the armpit on both sides of the mouse. Xenografts were examined every 3 days with a digital caliper and tumor volumes were calculated using the formula (length x width^2^)/2. After 27 days, mice were sacrificed, and tumor samples were embedded in paraffin for immunohistochemistry labeling using Ki-67 antibody, followed by hematoxylin and eosin staining.

### Cell proliferation, transwell migration and invasion assays

Cell proliferation test was performed using the WST-1 kit (Beyotime Biotechnology, Shanghai, China). NSCLC cells were plated in 96-well plates at a density of 1000 cells/well and cultured for 24 h. They were transfected with siRNA and further cultured for 96 h. The optical density was measured at 450 nm after adding 10 μL of WST-1 solution to 100 μL of RPMI-1640 medium.

Cell migration and invasion were determined by transwell migration assay and matrigel invasion assay (BD Falcon, San Jose, CA, USA), according to the manufacture’s instructions. Briefly, transwell migration assay was performed by suspending 5 × 10^4^ cells in 200 µL of serum-free RPMI-1640 medium and placing them in the cell culture insert (8 µm pore size) of a plate containing pre-warmed RPMI-1640 medium with 20% fetal bovine serum. Cells were cultured for 12 h and fixed with 4% paraformaldehyde. For matrigel invasion assay, 1 × 10^5^ cells were placed in the cell culture insert precoated with matrigel. Following addition of pre-warmed RPMI-1640 medium with 20% fetal bovine serum to the well, they were further cultured for 24 h and fixed with 4% paraformaldehyde. Cells were stained with 0.1% crystal violet and random regions were imaged using an optical microscope.

### Reverse transcription and quantitative PCR (RT-qPCR)

Total RNAs were extracted from cells or tissues using TRIzol reagent (TaKaRa, Dalian, China) according to the manufacturer’s instructions. Cytoplasmic and nuclear RNAs were prepared using the RNA Subcellular Isolation Kit (Active Motif) as described previously [7]. Reverse transcription of total RNAs (500 ng) was performed using the PrimeScript RT reagent Kit (TaKaRa, Dalian, China) in the presence of random primers. qPCR was performed using the Roche LightCycler^®^ 480 System and SYBR Premix Ex Taq (TaKaRa, Dalian, China), in the presence of gene-specific primers (Supplementary Table S2).

### Colony formation assay

Cells were digested with trypsin and seeded in 6-well plates with a density of 200 cells/well and cultured at 37 °C. After 10 days, the culture medium was removed, and cells were washed twice with PBS. They were fixed with methanol for 20 min, and colonies were stained with crystal violet for 20 min. After washing excess of crystal purple with PBS, the plates were dried and imaged for colony counting.

### Flow cytometry assays of apoptosis

Fluorescein Isothiocyanate (FITC) Annexin V Apoptosis Detection Kit (BD Biosciences) was used to detected apoptosis. After 48 h of transfection, cells were collected and washed with PBS. They were resuspended in the binding buffer and stained with 5 µL of Annexin V-FITC and propidium iodide in the dark for 15 min. Cells were sorted by flow cytometry and analyzed using the BD FACSDiva6.1 software (BD Biosciences).

### In situ hybridization

Lung tissues were fixed in 4% formaldehyde overnight at 4 °C, and paraffin sections of 10 µm were prepared using a microtome (Leica, RM2125RTS). Digoxigenin-labeled *LINC01279* probe was synthesized using the DIG RNA labeling Kit (Roche). After hybridization and incubation with alkaline phosphatase-conjugated anti-digoxigenin antibody (Roche), *LINC01279* signals were visualized using NBT/BCIP as a substrate.

### Western blot

Cells were lysed using the RIPA buffer in the presence of a mixture of protease inhibitors. Proteins were separated by SDS-PAGE and transferred to the polyvinylidene fluoride membrane. Non-specific antibody binding was blocked using 5% skimmed milk for 1 h, and incubation with primary antibodies (Supplementary Table S3) was performed at 4 °C overnight. After washing several times with TBST and incubation with secondary antibodies for 2 h, protein bands were detected using Luminate Western HRP substrates (Millipore) and Tanon 5200 chemiluminescence imaging system (Shanghai, China).

### RNA immunoprecipitation (RIP) assay

This was performed using the EZ-Magna RIP™ RNA-Binding Protein Immunoprecipitation Kit (Millipore). Cells were lysed in 100 µL of RIP lysis buffer containing protease inhibitor cocktail and DNase inhibitor. RNA–protein complexes were immunoprecipitated with SIN3A antibody (5 µg) or control IgG at 4 °C overnight. The magnetic beads were washed 6 times in washing buffer, and proteins were digested using protease K at 55 °C. Precipitated RNAs were analyzed by qPCR after reverse transcription in the presence of random primers.

### Analysis of fluorescent-tagged LC3 punctae

A tandem fluorescent-tagged LC3 construct was used to monitor the formation of GFP-LC3 and RFP-LC3 punctae [12]. Plasmids were transfected into H1299 and PC-9 cells using the Lipofectamine^®^ RNAiMAX Reagent (Invitrogen). After 48 h, cells were washed with PBS and incubated with Earle's balanced salt solution (E2888, Sigma) for an appropriate period. The formation of fluorescent punctae was visualized by confocal microscopy.

### Statistical analysis

All data were collected from three independent experiments. They were analyzed using GraphPad Prism (GraphPad Software, La Jolla, CA) and expressed as mean ± SD. Survival data were calculated using the Kaplan–Meier method and analyzed using the log-rank test. Unless specified, Student’s t-test was performed to determine statistical significance. *P* value < 0.05 was considered as statistically significant.

## Results

### *LINC01279* is highly expressed in LUAD

**Fig. 1:**
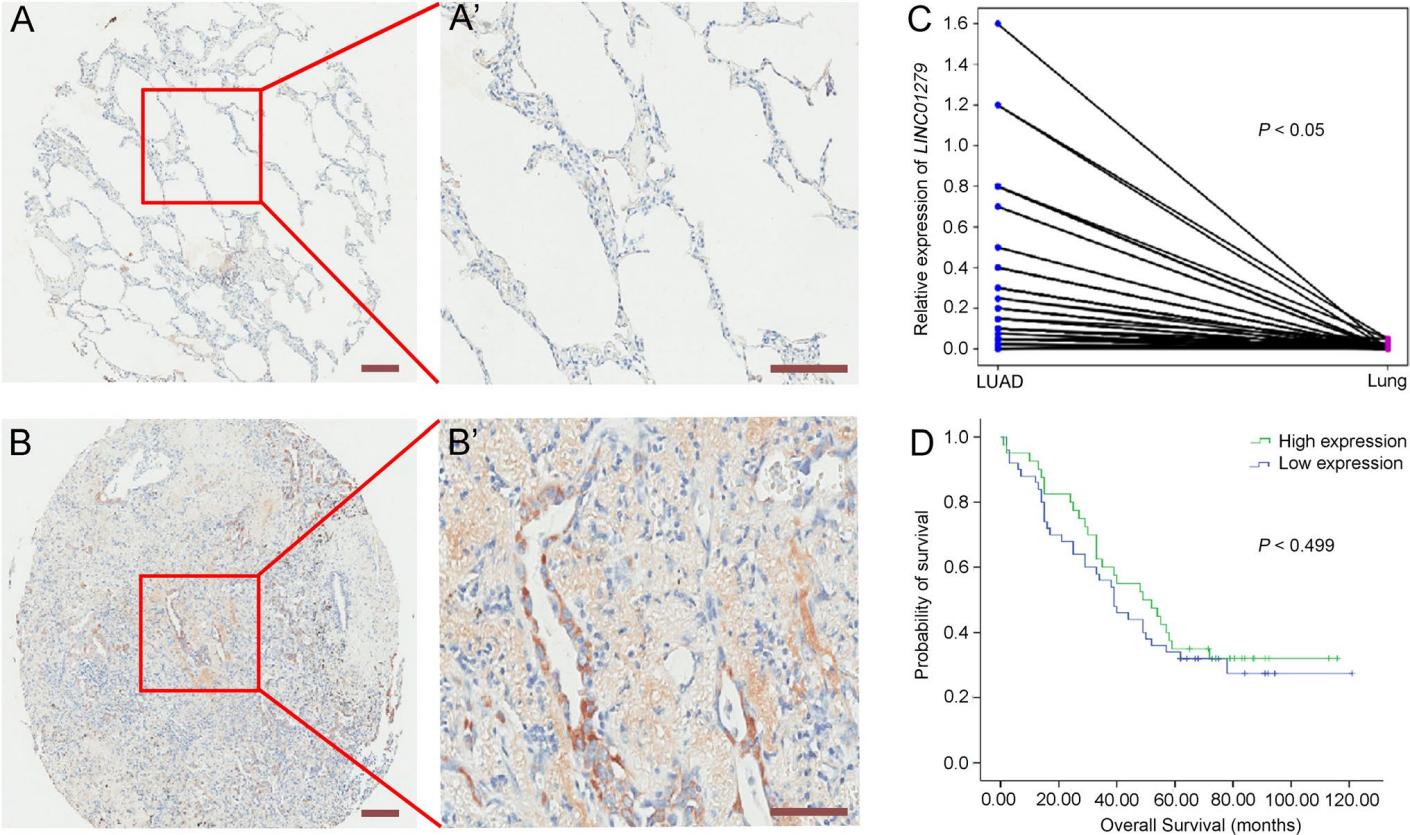
Up-regulation of *LINC01279* in LUAD. **A**-**B**’ In situ hybridization analyses compare the expression of *LINC01279* between noncancerous **A**, **A**’ and LUAD tissues **B**, **B**’. Note the presence of intense signals (brown colored) in LUAD tissues. **C** Analyses by RT-qPCR of the relative expression of *LINC01279* between LUAD and noncancerous lung tissues from a cohort of patients (*n* = 90; *, *P* < 0.05). **D** Analyses using the Kaplan–Meier method show the correlation of *LINC01279* expression levels with overall survival rates (cut-off at 50%). No significant differences in the probability of survival between groups with high and low expression of *LINC01279* (*P* = 0.499). Scale bars: 200 µm

We first used in situ hybridization to compare the expression of *LINC01279* in 90 pairs of non-cancerous and LUAD tissues. Compared with adjacent noncancerous (paracancer) lung tissues, which displayed clearly visible epithelia and lacked apparent *LINC01279* hybridization signal (Fig. 1A, A’), LUAD specimens showed strong *LINC01279* expression both in the lung epithelia and in the densely organized lung mesenchyme (Fig. 1B, B’). This observation suggests that *LINC01279* expression is associated with the process of LUAD. However, clinicopathological analyses indicated that *LINC01279* expression levels were not correlated with age of diagnosis, gender, and smoking status. We then analyzed *LINC01279* expression in the same LUAD tissues from 90 patients by qRT-PCR. The result indicated a significant up-regulation of *LINC01279* in LUAD tissues when compared with adjacent normal lung tissues (Fig. 1C), further demonstrating a close link between *LINC01279* expression and the occurrence or progression of LUAD. We next performed Kaplan–Meier survival analyses and log-rank tests to determine how *LINC01279* is related to the prognosis of LUAD. Using the HLugA180Su06 LUAD tissue microarray with associated survival information, we found that low and high expression of *LINC01279* (45 patients in each condition) were not correlated with reduced OS rates overtime (Fig. 1D). Consistently, correlation analysis from the TCGA database also shows a similar result. This implies that both high and low expression levels of *LINC01279* are correlated with the occurrence of LUAD, but they are not prognostic factors of this cancer.

### Knockdown of *LINC01279* prevents tumor progression and promotes apoptosis

**Fig. 2:**
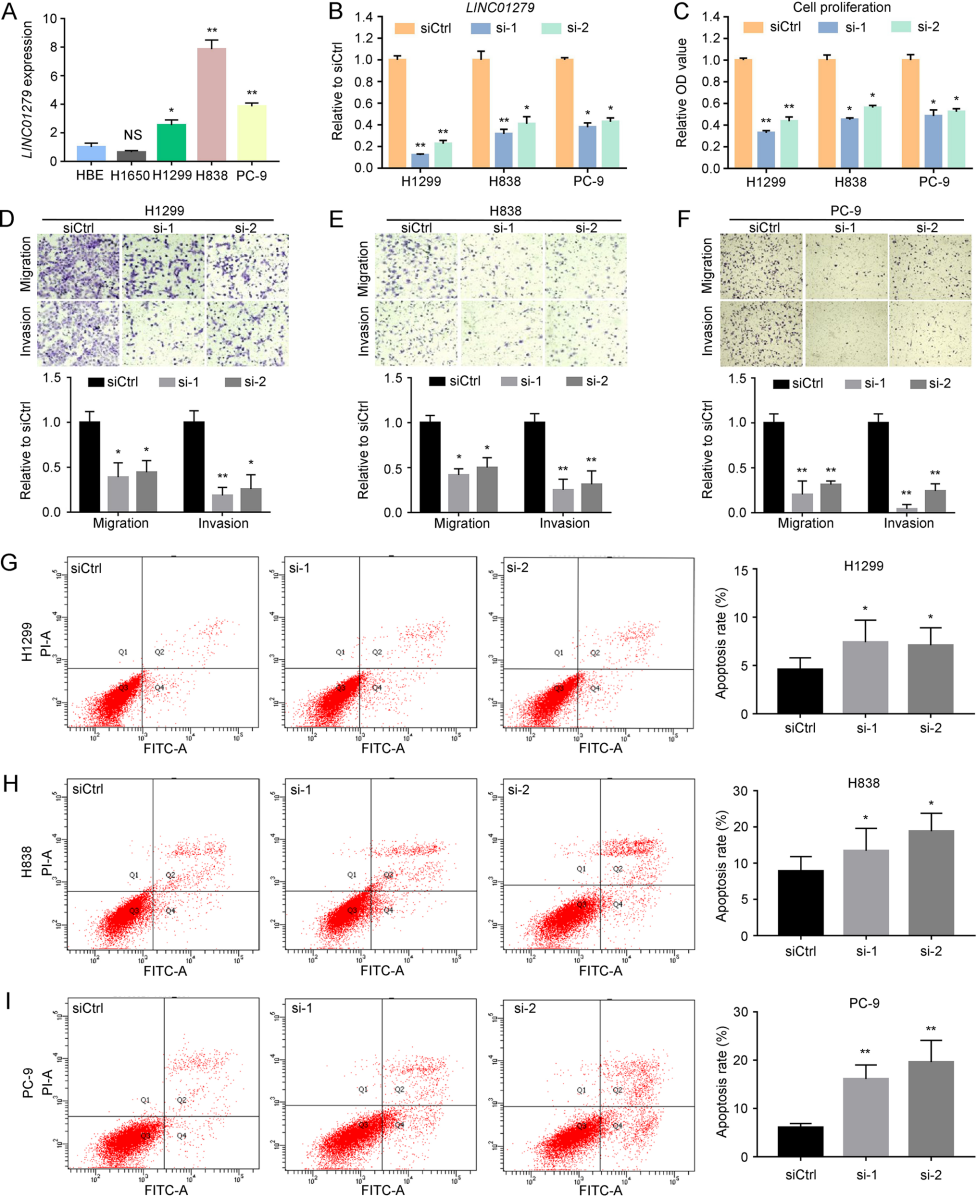
Knockdown of *LINC01279* inhibits tumor progression and promotes apoptosis. **A** RT-qPCR analyses show relative expression levels of *LINC01279* in HEB, H1650, H1299, H838 and PC-9 cells. The expression level of *LINC01279* in HEB is set to 1 as a reference, after normalization with GAPDH. **B** RT-qPCR analyses of *LINC01279* knockdown efficiency in H1299, H838 and PC-9 cells. These cells were treated with control siRNA (siCtrl) or two *LINC01279*-specific siRNAs (si-1 and si-2). The expression level of *LINC01279* in siCtrl-treated conditions is set to 1 as a reference, after normalization with GAPDH. **C** WST-1 assays show reduced cell proliferation following *LINC01279* knockdown in H1299, H838 and PC-9 cells. Values in siCtrl-treated conditions are set to 1 as a reference. **D**–**F** Knockdown of *LINC01279* reduces migration and invasion of H1299, H838 and PC-9 cells. **G**–**I** Flow cytometric analyses show that silencing of *LINC01279* promotes apoptosis of H1299, H838 and PC-9 cells. Statistical data are expressed as the mean ± s.e.m. from three independent experiments (*, *P* < 0.05; **, *P* < 0.01)

In order to understand the role of *LINC01279* in LUAD, we examined the effects of its knockdown in NSCLC cell lines. First, *LINC01279* expression levels were analyzed by RT-qPCR in H1299, H838, H1650 and PC-9 cells in comparison with the transformed normal bronchial epithelial (HBE) cells. Increased *LINC01279* expression was observed in H1299, H838 and PC-9 cells, but not in H1650 cells (Fig. 2A). Thus, H1299, H838 and PC-9 cells were used to analyze how *LINC01279* may be involved in cancer progression by small interfering RNA (siRNA) approach. Plasmids allowing the expressing of either a control siRNA (siCtrl) that should not target any sequence or two specific siRNAs targeting *LINC01279* (si-1 and si-2) were transfected into these cells. The silencing efficiency was determined by qRT-PCR, which showed strongly decreased expression of *LINC01279* in all three cell lines following knockdown by either si-1 or si-2 (Fig. 2B). WST-1 colorimetric assays indicated that knockdown of *LINC01279* significantly inhibited cell proliferation (Fig. 2C), and colony formation assay also showed strongly decreased clonogenic survival (Supplementary Fig. S1A-B). These results imply that *LINC01279* should normally function to promote the proliferative activity and progression of LUAD. We further analyzed how *LINC01279* regulates cancer progression using transwell migration and invasion assays. The results clearly indicated that knockdown of *LINC01279* significantly reduced the migratory ability and invasion capability of H1299, H838 and PC-9 cells (Fig. 2D–F). Moreover, this reduced tumor progression was correlated with an increased apoptosis (Fig. 2G-I), as determined by flow cytometry assays after staining with fluorescent isothiocyanate (FITC)-labeled annexin V and propidium iodide (PI). Together, these observations suggest that inhibition of *LINC01279* function may prevent cancer progression by reducing cell proliferation and inducing apoptosis.

**Fig. 3:**
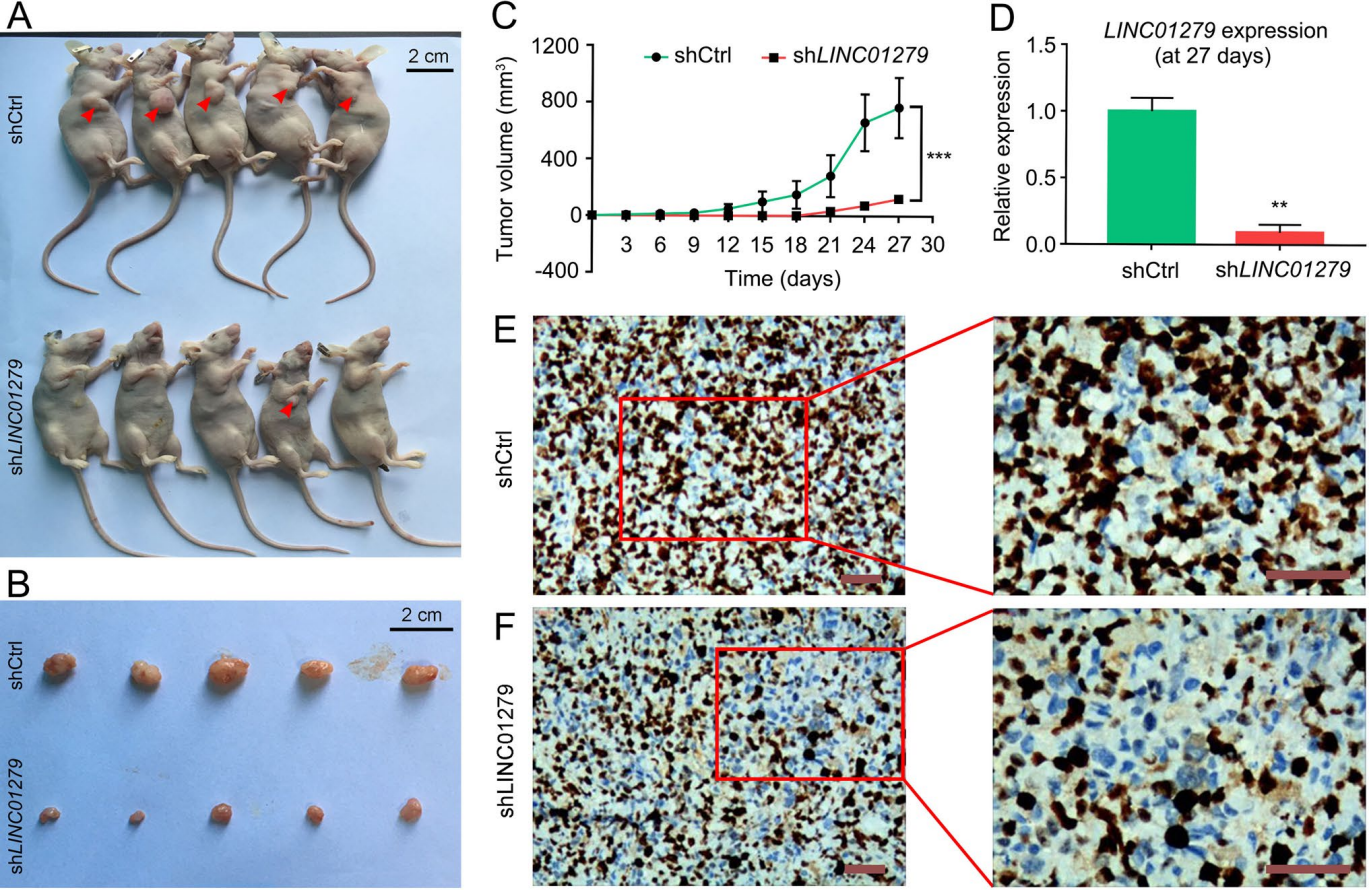
Knockdown of *LINC01279* prevents tumor growth in xenografts. H1299 cells were stably transfected with control shRNA (shCtrl) or sh*LINC01279*, and inoculated into nude mice to induce the formation of subcutaneous xenograft tumors (*n* = 5 for each condition). **A** External aspects of tumor growth (arrowheads) in mice at 27 days following implantation of shCtrl- and sh*LINC01279*-transfected cells. **B** Comparison of tumor size between shCtrl and sh*LINC01279* groups at 27 days after implantation. (C) Analyses of tumor growth rate overtime. Values were calculated using 5 animals for each condition (***, *P* < 0.001). **D** RT-qPCR analyses show strongly reduced expression of *LINC01279* in sh*LINC01279*-treated tumor tissues. The expression level of *LINC01279* in shCtrl-treated xenografts is set to 1 as a reference, after normalization with GAPDH. Data are the mean ± s.e.m. from three independent experiments (**, *P* < 0.01). **E**, **F** Reduced cell proliferation of sh*LINC01279*-treated tumor tissues, as revealed by immunohistochemistry of the cell proliferation antigen Ki-67. Scale bars: 100 µm

The tumor-inhibiting effects following *LINC01279* knockdown were further confirmed using xenografts derived from NSCLC cell lines. H1299 cells were stably transfected with plasmids allowing the expression of a double stranded short hairpin RNA (shRNA) targeting *LINC01279* (sh*LINC01279*) or a control shRNA that is not expected to recognize any known genes (shCtrl). They were then implanted into nude mice, and xenografts were examined every 3 days. Most obviously, at 27 days, mice implanted with xenografts expressing the shCtrl developed large-sized tumors, whereas mice carrying xenografts expressing the sh*LINC01279* displayed no visible or small-sized tumors (Fig. 3A, C). The inhibition of tumor growth is tightly correlated with a strongly decreased *LINC01279* expression level and reduced cell proliferation, as determined by qRT-PCR analysis and by immunocytochemical staining of the cell proliferation antigen Ki-67, respectively (Fig. 3D–F). Thus, results from our in vivo analyses strongly implicate *LINC01279* in the oncogenesis of LUAD; they demonstrate that suppression of *LINC01279* can efficiently prevent LUAD progression.

### *LINC01279* regulates FAK/ERK signaling in LUAD cell lines

**Fig. 4:**
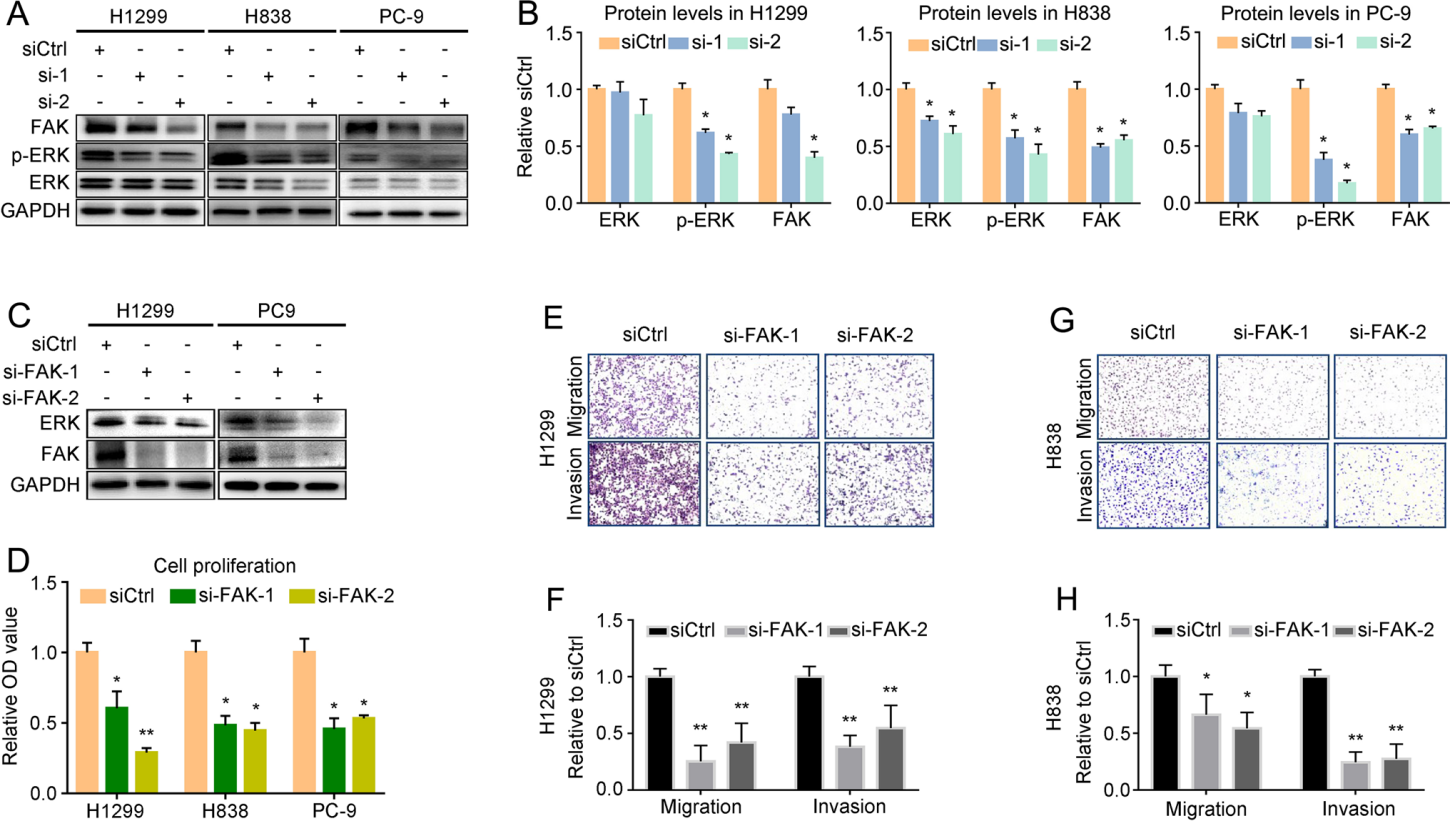
*LINC01279* regulates FAK and p-ERK levels. **A** Western blot analyses show that knockdown of *LINC01279* reduces FAK and p-ERK levels in H1299, H838 and PC9 cells. Total ERK protein levels showed no consistent changes following *LINC01279* knockdown. **B** Quantification of ERK, p-ERK and FAK protein levels. Values in siCtrl-treated conditions are set to 1 as a reference, after normalization with GAPDH. Data are the mean ± s.e.m. from three independent experiments (*, *P* < 0.05). **C** Western blot analyses show that knockdown of FAK reduces total ERK protein levels in H1299 and PC-9 cells. **D** Reduced proliferation of NSCLC cells following FAK knockdown, as determined by WST-1 assays. **E**–**H** Silencing of FAK reduces migration and invasion of H1299 and H838 cells. Values in siCtrl-treated conditions are set to 1 as a reference. Data are expressed as the mean ± s.e.m. from three independent experiments (*, *P* < 0.05; **, *P* < 0.01)

We next set to investigate the mechanisms underlying *LINC01279* function in the oncogenesis of LUAD. Analysis by qRT-PCR indicated that *LINC01279* was mostly enriched in the cytoplasm of H1299, H838 and PC-9 cells, representing 55% to 75% of the total amount (Supplementary Fig. S2). This suggests that it may function predominantly in the cytoplasm, but should also have activity in the nucleus. To explore *LINC01279*-regulated events in LUAD, we used a candidate approach to analyze expression changes of several signaling proteins potentially involved in tumorigenesis, including focal adhesion kinase (FAK) and extracellular signal-regulated kinase (ERK), which have established roles in LUAD. Following *LINC01279* knockdown in H1299, H838 and PC-9 cells, western blot analyses showed that protein levels of FAK were significantly reduced in different NSCLC cell lines (Fig. 4A, B). Protein level of ERK showed no changes in H1299 and PC-9 cells, although a moderately decrease was observed in H838 cells. However, phosphorylated ERK (p-ERK) levels were significantly reduced in all these cell lines (Fig. 4A, B). This may be secondary to decreased FAK expression because there are many lines of evidence indicating FAK-dependent regulation of ERK signaling [13–16]. Moreover, qRT-PCR analyses showed that knockdown of *LINC01279* did not inhibit the expression of FAK or ERK mRNAs (Supplementary Fig. S3). These observations suggest that *LINC01279* may regulate the translation and/or activity of FAK in NSCLC cells. Indeed, aberrant activation of FAK and ERK has been associated with dysregulated cell proliferation and migration in most cancer types [17–19]. Moreover, using public Web tools (http://kmplot.com/), we found that high expression of ERK protein was associated with poor survival prognosis in patients with LUAD (Supplementary Fig. S4). Thus, there is a possibility that *LINC01279* promotes tumorigenesis at least by indirectly activating the ERK pathway.

To examine whether FAK displays similar functions as *LINC01279* in LUAD, we designed two siRNAs targeting FAK (si-FAK-1 and si-FAK-2) to inhibit its expression in H1299 and PC-9 cells. Western blot analyses showed a significant decrease of FAK protein levels, suggesting efficient knockdown (Fig. 4C, Supplementary Fig. S5A, B). Interestingly, total ERK protein levels were also decreased in FAK knockdown cells (Fig. 4C, Supplementary Fig. S5A, B). We thus performed qRT-PCR analyses to determine how FAK regulates ERK, and found that there was a strongly reduced expression of ERK mRNA in H1299, H838 and PC-9 cells (Supplementary Fig. S5C). This suggests that a FAK-mediated pathway is required for the expression of ERK either at transcriptional or at post-transcriptional level, further supporting the conclusion that FAK functions upstream of ERK and that the reduced p-ERK levels following *LINC01279* knockdown in LUAD cells may be mediated by FAK. However, we cannot exclude the possibility that *LINC01279* also regulates ERK signaling at the translation level, because its knockdown did not affect the expression of ERK mRNA (Supplementary Fig. S3). As *LINC01279*, knockdown of FAK inhibited proliferation, cell cycle progression, migration and invasion of LUAD cell lines (Fig. 4D–H, Supplementary Fig. S6). Thus, these observations raise the possibility that *LINC01279* exerts its oncogenic activity at least partially through regulation of FAK protein synthesis and/or stability.

### Knockdown of *LINC01279* and FAK induces apoptosis of NSCLC cells

**Fig. 5:**
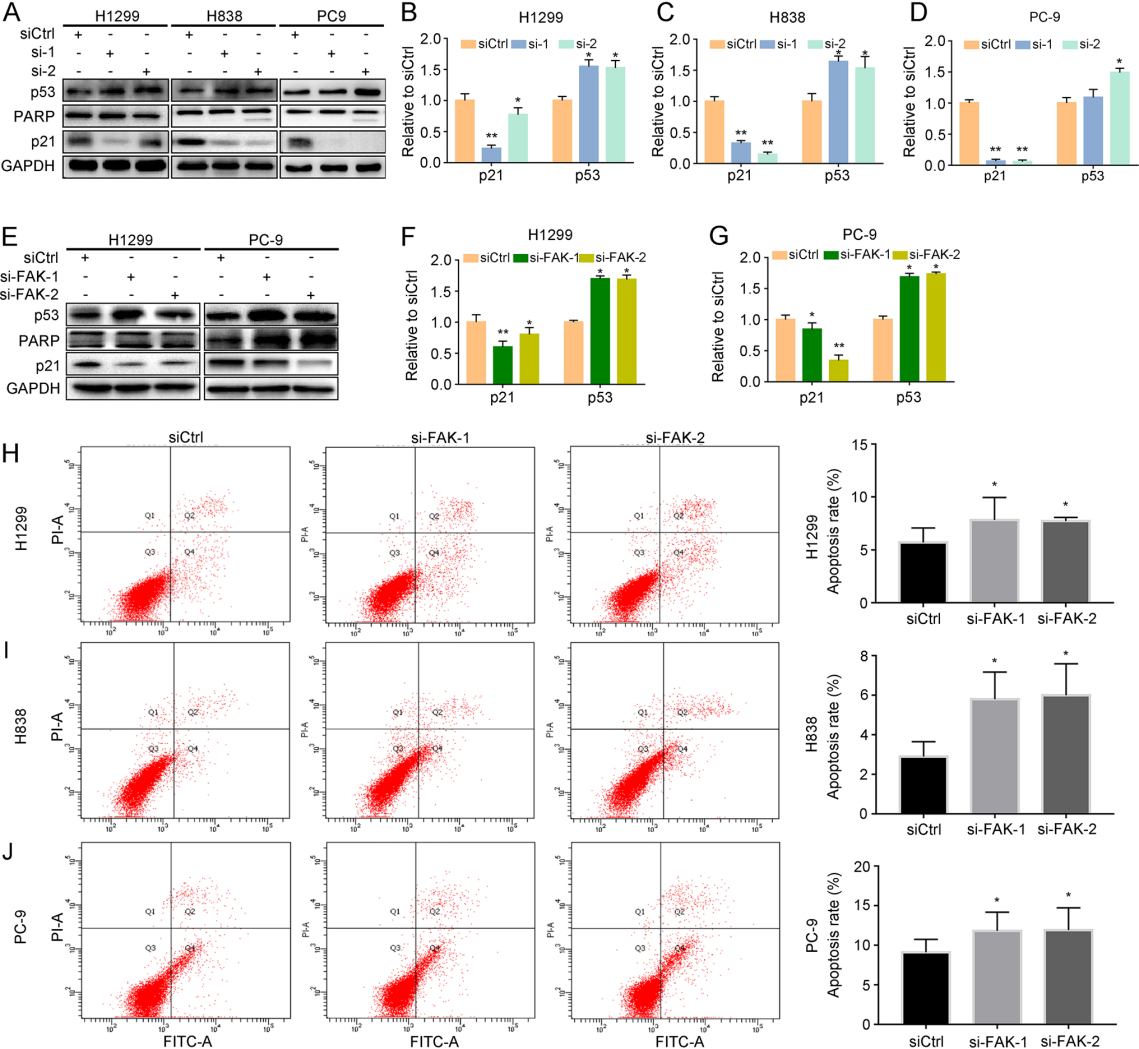
Knockdown of *LINC01279* and FAK induces apoptosis in NSCLC cells. **A** Western blot analyses of p53, p21 and PARP proteins after knockdown of *LINC01279* in H1299, H838 and PC9 cells. Cleaved fragments from PARP proteins can be detected in H838 and PC9 cells (lower bands in si-2-treated conditions), but not in H1299 cells. **B**–**D** Quantification of western blots shows increased p53 expression but decreased p21 levels after knockdown of *LINC01279*. **E** Western blot analyses of p53, p21 and PARP proteins following knockdown of FAK in H838 and PC9 cells. **F**, **G** Quantification of p53 and p21 protein levels. (**H**-**J**) Silencing of FAK promotes apoptosis in H1299, H838 and PC9 cells, as determined by flow cytometric assays. Statistical data are expressed as the mean ± s.e.m. from three independent experiments (*, *P* < 0.05; **, *P* < 0.01)

Abnormal apoptosis is related to malignant transformation, tumor metastasis, and resistance to anticancer drugs [20]. It is well established that p53 functions as a tumor suppressor and inhibits tumor growth by inducing apoptosis [21–23]. Although the cell cycle inhibitor p21 generally acts as target of p53 to induce apoptosis [24], there is also evidence that it functions as an inhibitor of apoptosis and displays oncogenic activity in a number of cancers [25–27]. In particular, it has been shown that activation of p21 could accelerate lung metastasis [28]. Since knockdown of *LINC01279* induces apoptosis in NSCLC cells (see Fig. 2G-I), we sought to investigate possible changes of these apoptosis-related proteins by western blot analyses. The expression of p53 was increased, while the expression of p21 was decreased in H1299, H838 and PC-9 cells 72 h after *LINC01279* knockdown (Fig. 5A-D). This observation is consistent with an oncogenic role of p21 in several tumors, which may be dependent on its cytoplasmic localization and post-translational modifications such as phosphorylation [28]. Moreover, in H838 and PC-9 cells transfected with the *LINC01279* siRNA construct, cleavage of the chromatin-associated poly (ADP-ribose) polymerase (PARP) that produced a fragment of 89 kDa could be also detected, indicative of cell death (Fig. 5A, lower bands in PARP panel). Knockdown of FAK also increased p53 expression and decreased p21 expression in H1299 and PC-9 cells (Fig. 5E–G). Analyses by flow cytometry showed that suppression of FAK function led to significantly increased apoptosis of NSCLC cells (Fig. 5H–J). Given that knockdown of *LINC01279* reduces FAK protein levels (see Fig. 4A, B), it is likely that FAK may at least partially mediate the function of *LINC01279* to regulate apoptosis in NSCLC cells. This is consistent with the functional interaction of FAK with p53 and with the apoptosis-promoting effects of FAK inhibitors [29]. Moreover, since FAK plays a critical role in integrin-mediated cell adhesion and migration of cancer cells but also facilitates their survival under stress conditions [29], overexpression of *LINC01279* in LUAD may promote metastasis and cell survival by increasing FAK-regulated adhesion signaling and cell migration.

### *LINC01279* interacts with SIN3A protein

**Fig. 6:**
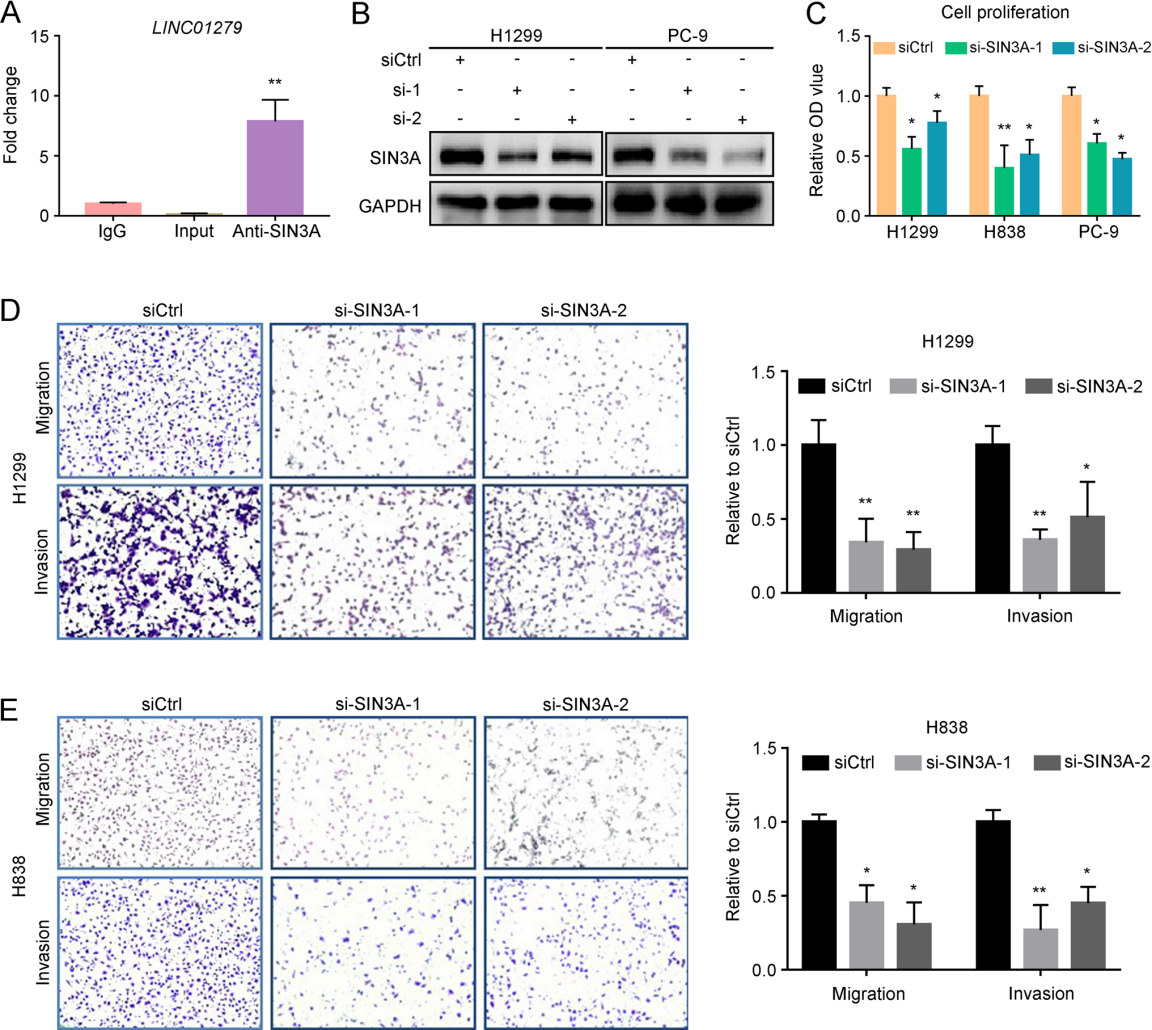
*LINC01279* interacts with and regulates the expression of SIN3A protein. **A** RIP-qPCR assays show the interaction between *LINC01279* and SIN3A protein. Samples were divided into two equal parts, which were processed for RNA purification in the presence of anti-SIN3A antibody or control IgG. The value obtained after PCR amplification from the control IgG sample is set to 1 as a reference. **B** Western blot analyses of SIN3A protein levels after knockdown of *LINC01279* in H1299 and PC-9 cells. **C** WST-1 assays show reduced cell proliferation following SIN3A knockdown in H1299, H838 and PC-9 cells. **D**, **E** Knockdown of SIN3A reduces migration and invasion of H1299 and H838 cells. Values in siCtrl-treated conditions are set to 1 as a reference. Data are expressed as the mean ± s.e.m. from three independent experiments (*, *P* < 0.05; **, *P* < 0.01)

To gain further insight into the mechanism by which *LINC01279* functions in LUAD, we searched the public database (https://www.genecards.org/) for its interacting proteins. Among several possible candidates, SIN3A was particularly interesting given its established role in tumorigenesis [30–32]. We thus experimentally verified the physical interaction by RIP-qPCR. The results clearly showed that *LINC01279* transcripts were significantly enriched by SIN3A-specific antibody (Fig. 6A), suggesting complex formation between SIN3A and *LINC01279*. We then asked the question whether this interaction mutually modulates the expression or stability of SIN3A and *LINC01279*. Two siRNAs (si-SIN3A-1 and si-SIN3A-2) designed against SIN3A significantly inhibited the expression of SIN3A at mRNA and protein levels, suggesting efficient knockdown (Supplementary Fig. S7A, B). However, this had no obvious effects on *LINC01279* expression in different NSCLC cell lines (Supplementary Fig. S7C). Conversely, knockdown of *LINC01279* decreased SIN3A protein expression, but not overall SIN3A transcript levels in H1299 and PC-9 cells (Fig. 6B, Supplementary Fig. S7D). These observations suggest that *LINC01279* may play a role in the stabilization of SIN3A, uncovering a novel role for this lncRNA in NSCLC cells. Consistent with its regulation by *LINC01279*, knockdown of SIN3A also reduced proliferation, migration and invasion of NSCLC cells (Fig. 6C–E), and caused increased apoptosis of these cells (Supplementary Fig. S8).

### Suppression of *LINC01279* and SIN3A induces autophagy

SIN3A plays an important role in cancer pathogenesis [30, 31] and functions as a global transcription regulator with diverse chromatin-modifying activities [33]. Nevertheless, how it modulates metastatic progression or apoptosis in lung cancer remains largely unclear. Previous studies showed that SIN3A is involved in regulating the expression of autophagy-related proteins in C2C12 myoblasts [34]. It is well established that potential pathways linking apoptosis and autophagy exist in cancer [35]. Since inhibition of either *LINC01279* or SIN3A leads to increased apoptosis, we compared the effects of their knockdown in initiating autophagy of NSCLC cells.

**Fig. 7:**
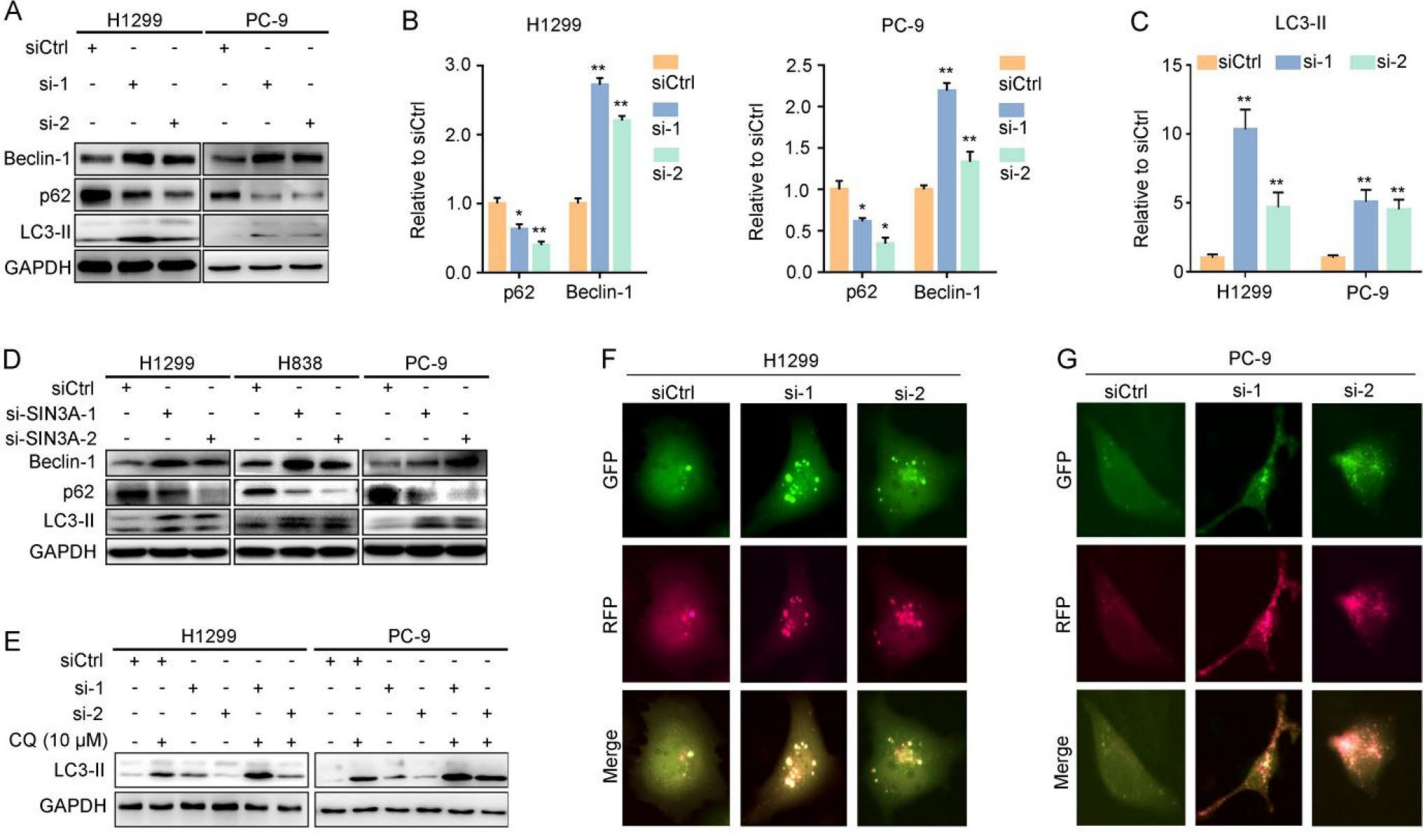
Knockdown of LINC01279 and SIN3A induces autophagy. **A**–**C** Western blot analyses and quantification of autophagy-related proteins after knockdown of *LINC01279* in H1299 and PC-9 cells indicate activation of autophagy. Note the increased expression of LC3-II and Beclin-1, and decreased expression of p62 in *LINC01279* knockdown conditions. Protein levels in siCtrl-treated conditions are set to 1 as a reference, after normalization with GAPDH. Data are the mean ± s.e.m. from three independent experiments (*, *P* < 0.05; **, *P* < 0.01). **D** Western blot analyses of autophagy-related proteins show that knockdown of SIN3A similarly activates autophoagy in H1299, H838 and PC-9 cells. **E** Western blot analyses show further increase of LC3-II accumulation following knockdown of *LINC01279* and treatment with chloroquine (CQ), suggesting enhanced autophagy flux. **F**, **G** Representative images show the formation of autophagosmes after knockdown of *LINC01279* in H1299 and PC-9 cells

The adaptor protein p62 is constantly subjected to degradation by non-selective autophagy from the formation of autophagosomes to the maturation of autophagolysosomes [36]. Thus, its accumulation inhibits autophagy, while its decreased expression produces the opposite effect [37]. During the initiation of autophagy, LC3-I is cleaved and lipidated to generate LC3-II, which in turn is recruited to autophagosomal membranes. Thus LC3-II can be used as a protein marker for the formation of autophagosomes [38]. Beclin1 (the mammalian ortholog of yeast Atg6) exhibits an evolutionarily conserved role in the formation of autophagosome and functions to inhibit tumor growth [39]. It also acts as a dual regulator and links autophagy to cell death [40]. Western blot analyses indicated that knockdown of *LINC01279* in H1299 and PC-9 cells significantly decreased p62 protein level but increased the expression of Beclin-1 protein (Fig. 7A, B). We also observed an increased production of LC3-II in both cell lines (Fig. 7A, C). These indicate that suppression of *LINC01279* can activate autophagy. Consistent with its interaction with *LINC01279*, knockdown of SIN3A had very similar effects on the expression of Beclin-1, p62 and LC3-II in H1299, H838 and PC-9 cell lines (Fig. 7D, Supplementary Fig. S9A-C). Given the regulation of SIN3A expression by *LINC01279*, these results suggest that they likely function in the same pathway to modulate autophagy-linked apoptosis in LUAD.

We next analyzed the accumulation of LC3-II following *LINC01279* knockdown in the presence of chloroquine, which is a known autophagy inhibitor that prevents the fusion of autophagosomes with lysosomes [41]. A further increase in LC3-II accumulation is indicative of enhanced autophagy flux [42]. Indeed, knockdown of *LINC01279* in H1299 and PC-9 cells in the presence of chloroquine (10 µM) further increased LC3-II protein levels (Fig. 7E, Supplementary Fig. S9D, E). Thus, this result suggests that suppression of *LINC01279* promotes autophagy and leads to apoptosis. To further address the effects of *LINC01279* knockdown on autophagy, we used a tandem fluorescent-tagged LC3 to monitor the formation of GFP-LC3 and RFP-LC3 punctae [12], which are positively correlated with autophagosome numbers in the cell [41]. Knockdown of *LINC01279* in H1299 and PC-9 cells led to obviously increased fluorescent punctae (Fig. 7F, G). Taken together, our results indicate that *LINC01279* plays a role in autophagy and suggest that it should normally function to inhibit apoptosis in LUAD cells at least in part by regulation of SIN3A protein expression.

## Discussion

There is increasing evidence suggesting that dysregulation of lncRNAs occurs in various diseases and critically contributes to cancer development [43, 44]. In this work, we have demonstrated that *LINC01279* is up-regulated in LUAD and is associated with cancer progression. It is involved in the migration and invasion of NSCLC cells through regulation of FAK and ERK signaling. Furthermore, we also found that it complexes with the tumor suppressor transcriptional co-repressor SIN3A and regulates the expression of this protein. Knockdown of *LINC01279* and SIN3A inhibited cell proliferation by inducing autophagy and apoptosis. Importantly, inhibiting the function of *LINC01279* prevented tumor progression in xenografts derived from NSCLC cell lines. These observations strongly implicate *LINC01279* in lung cancer development, making it a potential target for diagnosis and treatment of this cancer.

Based on the results from knockdown approaches using cultured NSCLC cell lines and xenografts derived from these cells, we conclude that increased expression of *LINC01279* promotes LUAD oncogenesis by regulating several proteins involved in cell migration and proliferation. Analyses of *LINC01279*-depedent protein expression changes indicate that FAK and ERK signaling pathways are possible targets of *LINC01279* in LUAD cells. FAK is a non-receptor tyrosine kinase highly expressed in cancer. It acts as a multi-functional regulator of cell signaling in the tumor microenvironment and controls cell migration and invasion through kinase-dependent and -independent mechanisms [29, 45]. Knockdown of *LINC01279* decreased FAK protein levels but had no effect on the stability of FAK mRNA, thus it is likely that *LINC01279* modulates FAK expression through translation of the mRNA, thereby influencing integrin-mediated cell adhesion. ERK/MAPK (mitogen-activated protein kinase) signaling pathway has been also implicated in multiple cellular processes such as proliferation, migration and apoptosis. There are many lines of evidence indicating that FAK can mediate phosphorylation of ERK and activation of the ERK pathway [13–16]. We found that knockdown of *LINC01279* had little effect on the expression of total ERK protein but significantly reduced p-ERK level. However, knockdown of FAK led to reduced expression of ERK mRNA and protein in different NSCLC cell lines. These observations suggest that FAK should mediate the activity of *LINC01279* and functions upstream of ERK to regulate cancer cell proliferation and migration. Nevertheless, how does FAK regulate ERK expression requires further investigation.

SIN3A, a member of the SIN3 family, functions as a component of the histone deacetylase (HDAC) complex and regulates key cellular processes linked to cancer pathogenesis and progression [33]. However, the exact role of SIN3A in tumorigenesis remains elusive. Previous studies suggested that SIN3A could function as a tumor suppressor. It regulates the expression of several genes involved in cell invasion [30, 31]. Recent works showed that it cooperates with STAT3 to repress the transcription of tumor suppressor genes, thus interfering with its expression leads to transcriptional derepression, resulting in increased tumor cell death and reduced tumorigenic potential of anaplastic large-cell lymphoma [32]. This oncogenic activity of SIN3A is consistent with our present observations. We made the interesting finding that SIN3A protein associates with and is stabilized by *LINC01279*. Moreover, we found that knockdown of *LINC01279* or SIN3A in NSCLC cells similarly induced autophagy and led to apoptosis, suggesting that they normally function to inhibit these processes. Although future studies will be needed to determine how *LINC01279* regulates SIN3A protein level, the present results suggest that increased expression of *LINC01279* in LUAD likely prevents cell death-related autophagy through regulation of SIN3A protein stability.

It is well known that cancer cells exhibit autophagy-dependent cell death, which is linked to apoptosis [46]. In the present study, we showed that suppression of *LINC01279* enhanced autophagy flux since LC3-II level further increased in the presence of chloroquine. Therefore, inhibiting the activity of *LINC01279* can induce autophagy and apoptosis, thereby preventing progression of LUAD. By contrast, increased expression of this IncRNA should lead to inhibition of autophagy-dependent cell death, thereby promoting proliferation of LUAD. Thus, our results imply that *LINC01279* may normally function to promote cancer development by inhibiting autophagy-dependent cell death. Whether *LINC01279* directly regulates autophagy-related proteins still needs further investigations. However, we found that SIN3A displays a similar activity as *LINC01279* in regulating the expression of autophagy-related proteins. It is of note that autophagy exhibits dual functions during tumorigenesis, often in a stage-dependent manner. It may act as a tumor suppressor that inhibits the expression of oncogenic proteins at early stages or may exert pro-tumor activity by promoting tumor cell survival at advanced stages [47]. Since *LINC01279* interacts with SIN3A, its may exert pro-tumor activity through regulation of SIN3A expression. In this regard, it has been shown recently that the SIN3A transcriptional repressor complex interacts with STAT3 and is involved in silencing tumor repressor genes, thus promoting cell survival in different cancers [32]. Our results showing that knockdown of SIN3A activates autophagy and attenuates cell proliferation supports a pro-tumor activity of this epigenetic regulator.

The relationship between *LINC01279*-regulated autophagy and apoptosis remains unclear and needs further investigations. Knockdown of *LINC01279* increased p53 protein levels and cleavage of PARP, which are hallmarks of apoptosis. This may be both dependent and independent of autophagy-induced cell death. Indeed, there exists a close interplay between autophagy and apoptosis, which may be regulated by common signaling pathways [46]. Results from the present study suggest that *LINC01279* may be an important regulator of both processes. We found that either high or low expression of *LINC01279* is correlated with a reduced OS rate in patients with LUAD. There is also evidence that upregulation of *LINC01279* may be related to tumor invasion in gastric cancer [10]. Thus, it may represent a potential therapeutic target to prevent tumor growth. Indeed, we showed that suppression of *LINC01279* efficiently prevented tumor growth in xenografts derived from NSCLC cells.

## Conclusions

Our present work revealed that *LINC01279* is significantly up-regulated in clinical LUAD tissues and cultured NSCLC cell lines. It has the potential to promote NSCLC cell invasion by regulating FAK and ERK. We also demonstrated that it interacts with and stabilizes the epigenetic regulator SIN3A to regulate autophagy-dependent cell death. These findings provide insights into the function of *LINC01279* as a regulator of cell survival in LUAD, and identify *LINC01279* as a novel potential predictive molecular marker in this cancer. Thus, this work can be served as the foundations for the development of new diagnostic and therapeutic targets in various cancers.

### Supplementary Information

Additional file1 (DOCX 643 KB)

## Data Availability

All the data obtained and/or analyzed during the current study are available from the corresponding authors on reasonable request.
