# Supplementary material for: Suppression of the long non-coding RNA LINC01279 triggers autophagy and apoptosis in lung cancer by regulating FAK and SIN3A
Source: Discov Oncol. 2024 Jan 2;15:3. doi: 10.1007/s12672-023-00855-4 (PMC10761653; doi:10.1007/s12672-023-00855-4)
Supplement: Supplementary file 1 — Additional file1 (DOCX 643 KB) [file 12672_2023_855_MOESM1_ESM.docx]

**Suppression of the long non-coding RNA *LINC01279* triggers autophagy and apoptosis in lung cancer by regulating FAK and SIN3A**

Jiancong Wu^1#^, Xiaobi Huang^1#^, Xiaofang Li^2^, Honglian Zhou^1^, Xiaorao Chen^1^, Yongyang Chen^1^, Yudong Guo^1^, Jian Huang^3^, Hanqing Huang^3^, Zhong Huang^1^, Guoan Chen^4^, Zhixiong Yang^1^*, Jian Zhang^4^*, Wenmei Su^1,5^*

^1^Department of Pulmonary Oncology, Affiliated Hospital of Guangdong Medical University, Zhanjiang, 524000, China

^2^Center for Pathological Diagnosis and Research, Affiliated Hospital of Guangdong Medical University, Zhanjiang, 524000, China

^3^Department of Thoracic Surgery, Maoming People's Hospital, Maoming, 525000, China

^4^School of Medicine, Southern University of Science and Technology, Shenzhen 518055, China

^5^Guangdong Provincial Key Laboratory of Autophagy and Major Chronic Non-communicable Diseases, Affiliated Hospital of Guangdong Medical University, Zhanjiang, 524000, China

Running title: *LINC01279* regulates LUAD progression.

^#^Jiancong Wu and Xiaobi Huang contributed equally.

*Correspondence: Wenmei Su ([suwenmei123@hotmail.com](mailto:suwenmei123@hotmail.com)), Zhixiong Yang ([yangzhixiong068@126.com), and](mailto:yangzhixiong068@126.com) Jian Zhang ([jianzhang008@hotmail.com](mailto:jianzhang008@hotmail.com))

#
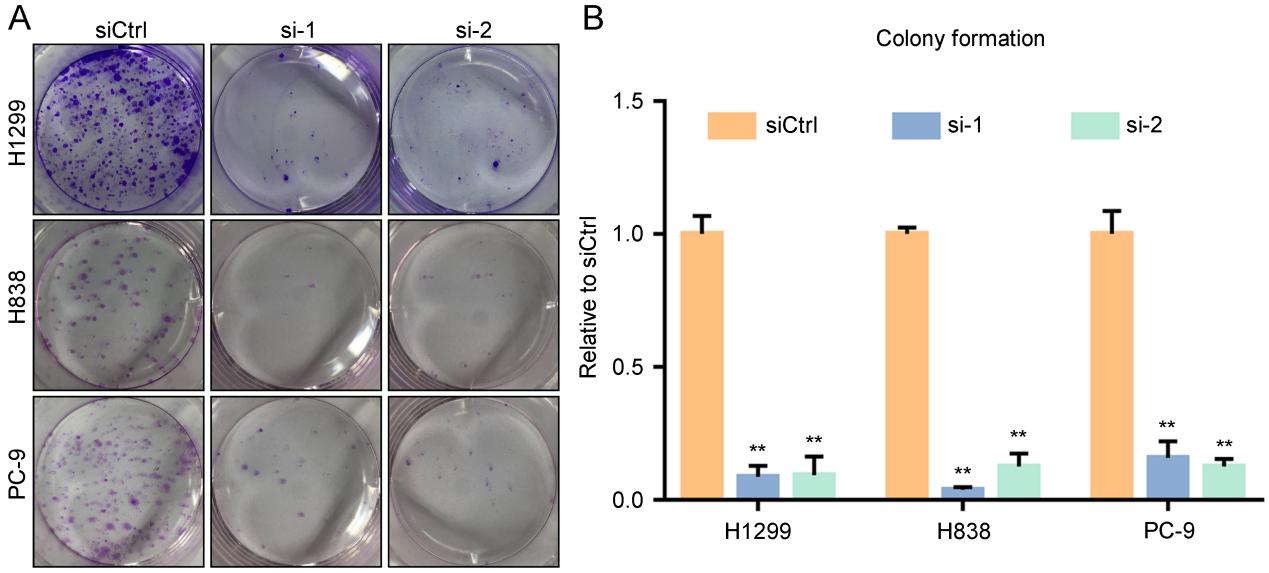


# Supplementary Figure S1. (A) Knockdown of *LINC01279* in H1299, H838 and PC-9 cells prevents colony formation. (B) Quantification of colony formation. Values were normalized to siCtrl-treated conditions. Data are the mean ± s.e.m. from three independent experiments (**, *P* <0.01).


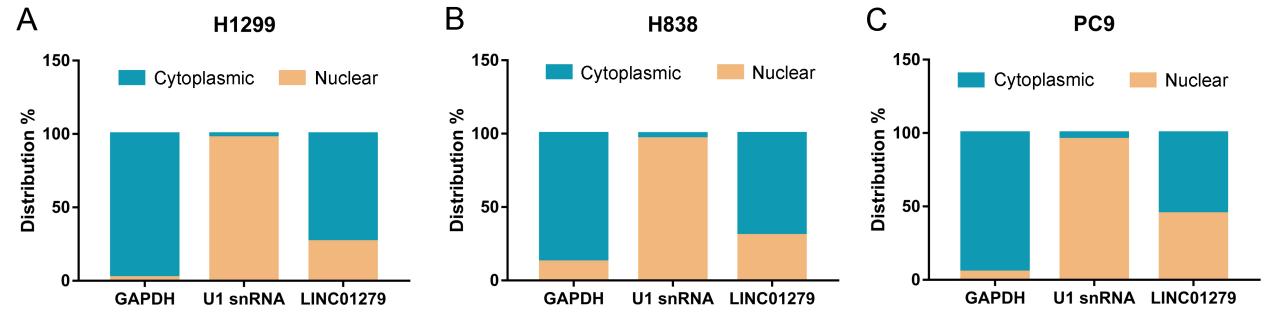


# Supplementary Figure S2. (A-C) RT-qPCR analyses of cytoplasmic and nuclear distributions of *LINC01279* in H1299, H838 and PC-9 cells. GAPDH and U1 snRNA were used as controls for the preparation of cytoplasmic and nuclear RNAs, respectively.


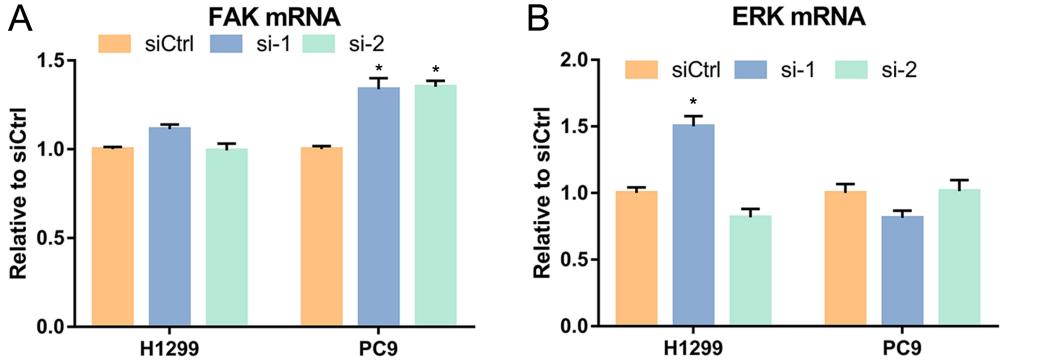


**Supplementary Figure S3.** (A, B) RT-qPCR analyses of the expression of FAK and ERK mRNAs after *LINC01279* knockdown in H1299 and PC-9 cells. Values were normalized to siCtrl-treated conditions. Data are the mean ± s.e.m. from three independent experiments (*, *P* < 0.05).


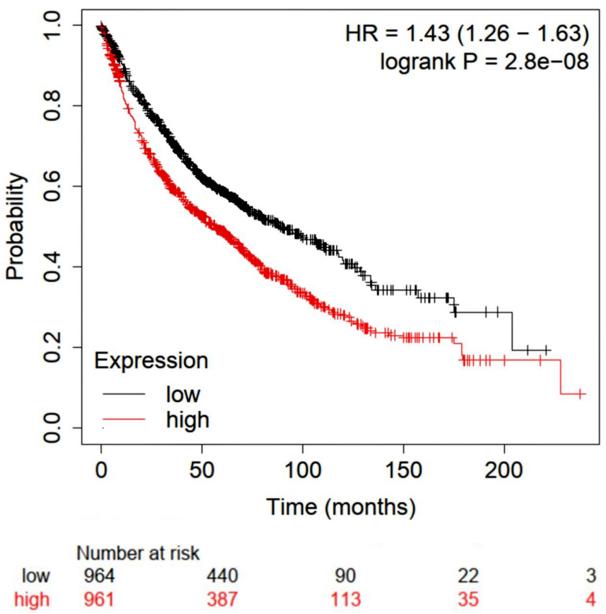


# Supplementary Figure S4. Correlation between ERK expression and probability of survival in patients with LUAD. Analysis using the Kaplan-Meier method (cut-off value at 50%) shows that high level of ERK expression is related to poor patient survival (HR=1.43, 1.26-1.63, logrank P= 2.8e-08).


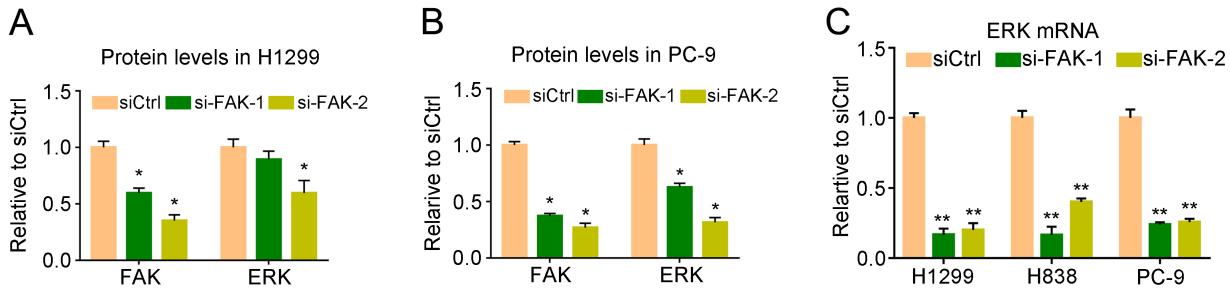


**Supplementary Figure S5.** Quantification of FAK and ERK protein levels and RT-qPCR analyses of ERK mRNA levels following FAK knockdown. (A, B) Knockdown of FAK in H1299 and PC-9 cells significantly decreases ERK proteins levels. (C) Knockdown of FAK significantly inhibits ERK mRNA levels in H1299, H838 and PC-9 cells. Statistical data are expressed as the mean ± s.e.m. from three independent experiments (*, *P* < 0.05; **, *P* < 0.01).


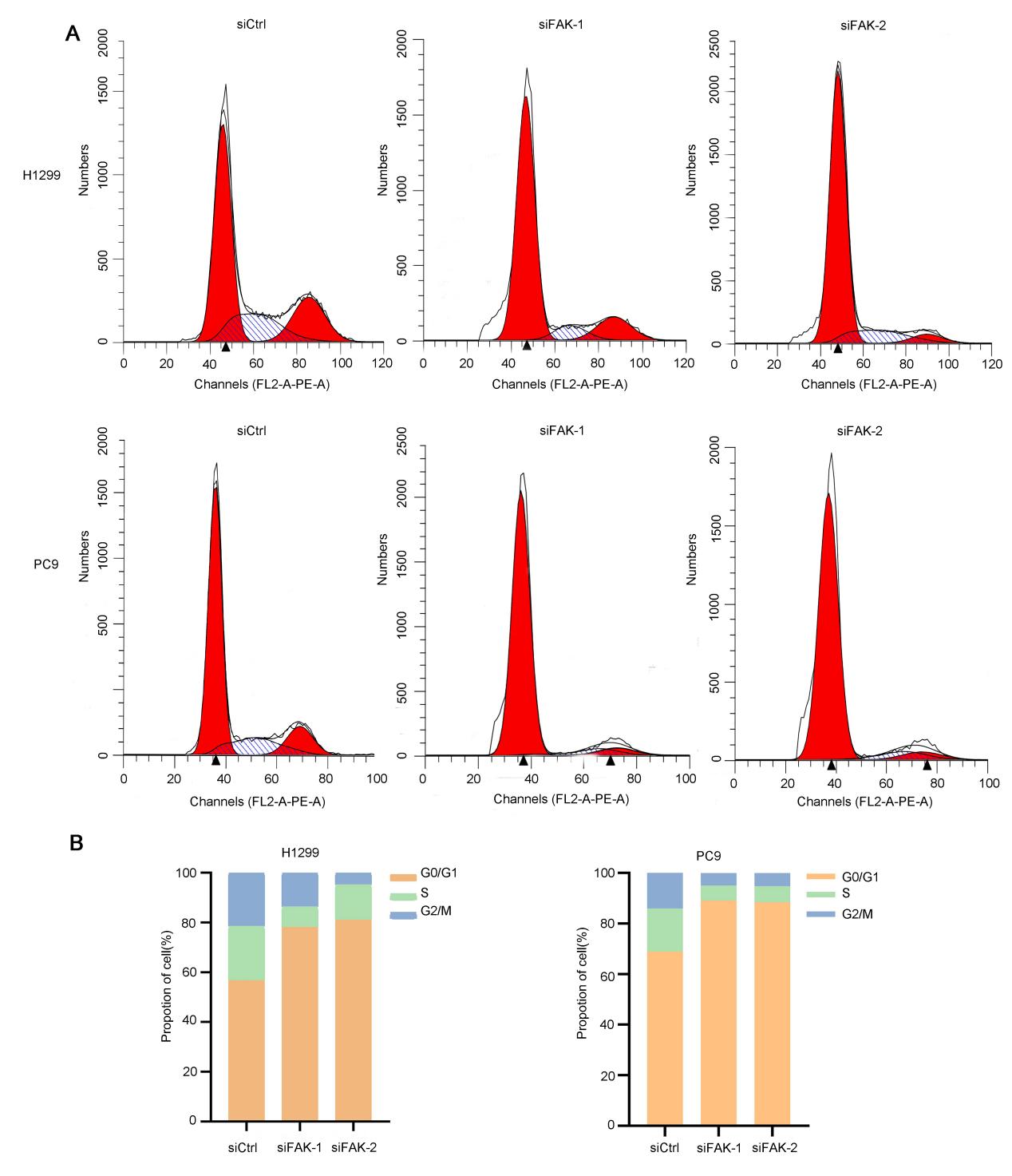


**Supplementary Figure S6.** Silencing FAK suppresses cell cycle progression in vitro. (A, B) Effects on cell cycle progression analyzed by flow cytometry after downregulation of FAK.


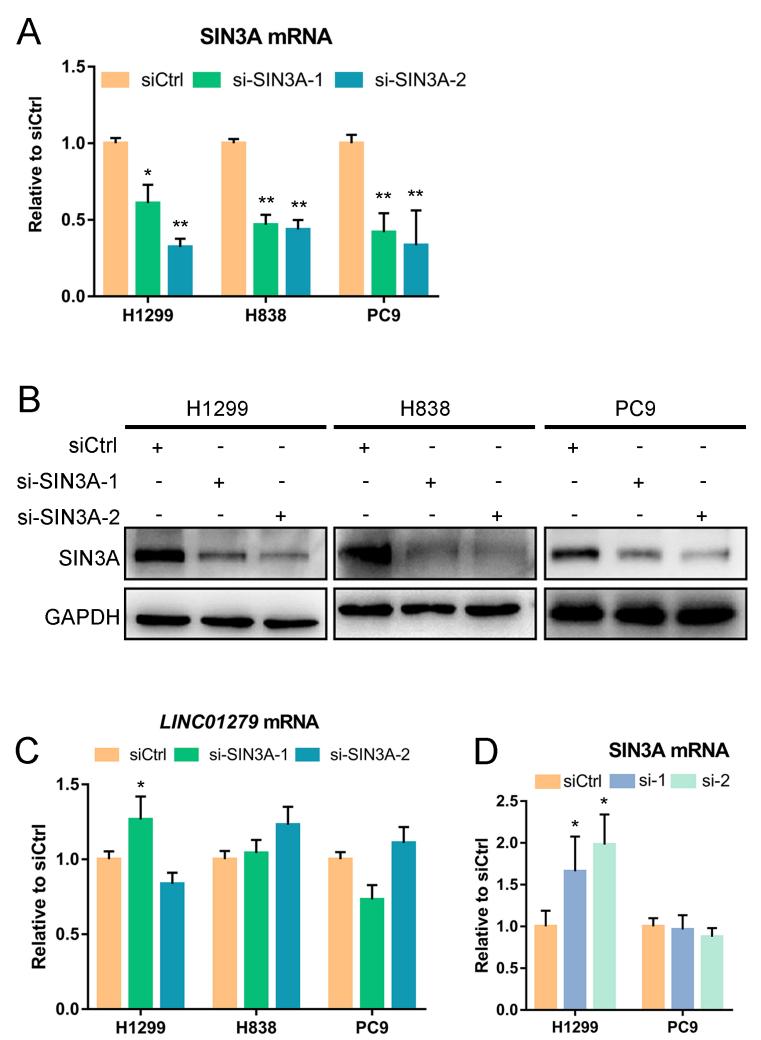


**Supplementary Figure S7.** Efficiency of SIN3A knockdown. (A) RT-qPCR analyses show the efficiency of SIN3A knockdown in H1299, H838 and PC-9 cells. (B) Western blot analyses show reduced SIN3A protein levels following SIN3A knockdown in NSCLC cell lines. (C) Knockdown of SIN3A in NSCLC cell lines has no significant effects on the expression of *LINC01279*. (D) Knockdown of *LINC01279* in H1299 and PC-9 cells does not reduce the expression of SIN3A mRNA.


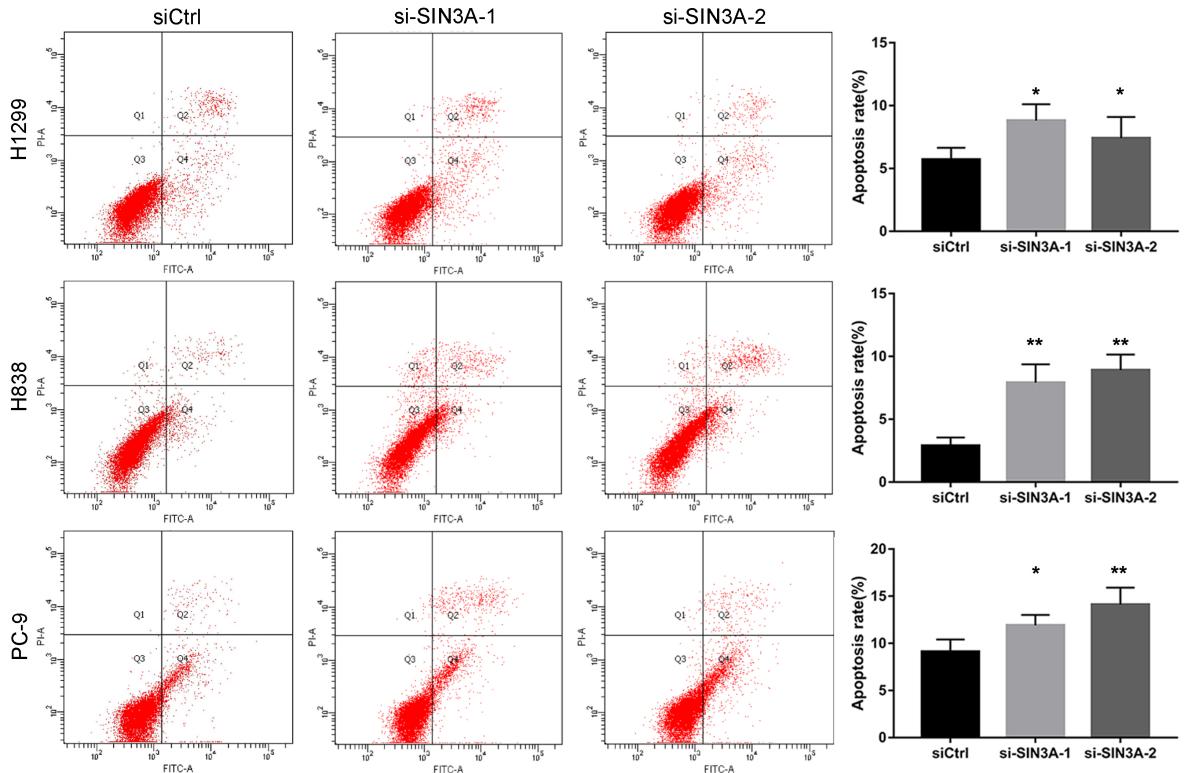


# Supplementary Figure S8. Knockdown of SIN3A induces apoptosis. Flow cytometry analyses show increased apoptosis following knockdown of SIN3A in H1299, H838 and PC-9 cells. Data are the mean ± s.e.m. from three independent experiments (*, *P* < 0.05; **, *P* < 0.01).


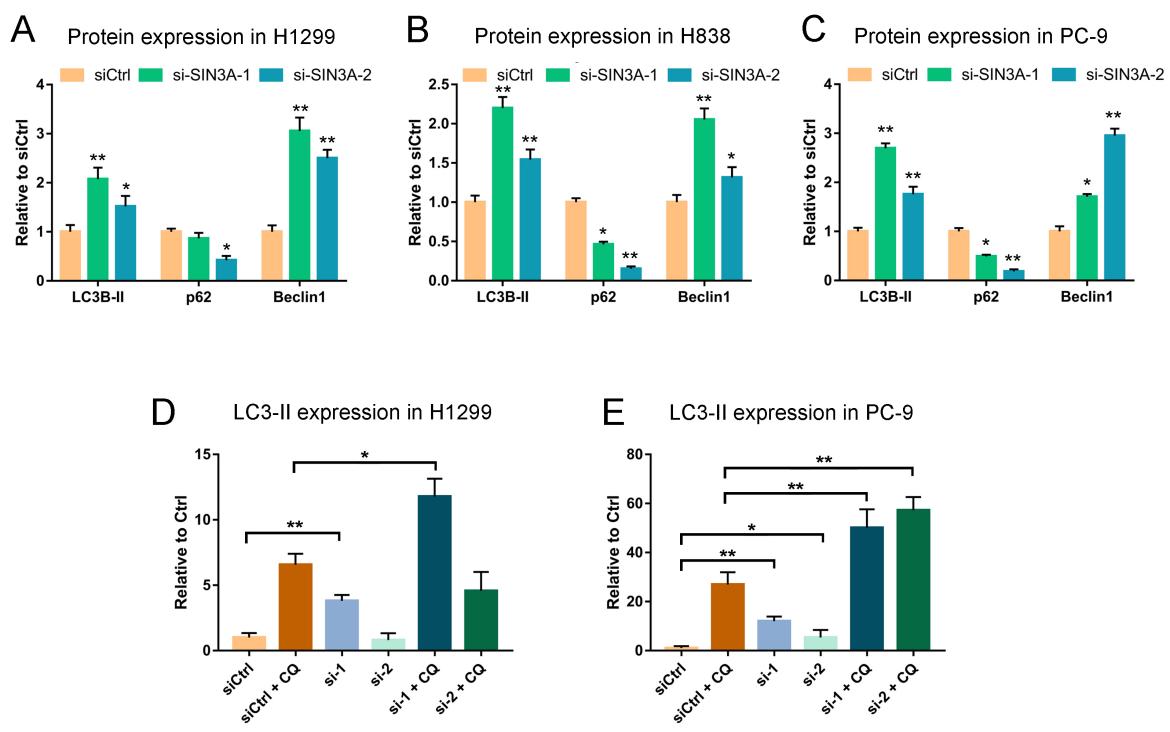


**Supplementary Figure S9.** Knockdown of *LINC01279* and SIN3A activates autophagy. (A-C) Quantification of LC3-II, p62 and Beclin-1 protein levels from western blot results shown in Figure 7D. (D, E) Increased autophagy flux following knockdown of *LINC01279*. Quantification of LC3-II levels from western blot results shown in Figure 7E. Statistical data are expressed as the mean ± s.e.m. from three independent experiments (*, *P* < 0.05; **, *P* < 0.01).

| Gene name | Sequences |
| --- | --- |
| LINC01279 #1 | 5’-GCCUGUACUUACUUGCUAATT-3’  5’-UUAGCAAGUAAGUACAGGCTT-3’ |
| LINC01279 #2 | 5’-UGAGAAAGAUUAUGGGAUUTT-3’  5’-AAUCCCAUAAUCUUUCUCATT-3’ |
| FAK #1 | 5’-GAACCUCGCAGUCAUUUAUTT-3’  5’-AUAAAUGACUGCGAGGUUCTT-3’ |
| FAK #2 | 5’-CUUUGGCGGUUGCAAUUAATT-3’  5’-UUAAUUGCAACCGCCAAAGTT-3’ |
| SIN3A #1 | 5’-GCAGUCAGCUACGGGAAUUTT-3’  5’-AAUUCCCGUAGCUGACUGCTT-3’ |
| SIN3A #2 | 5’-CCUCAGGUCUACAAUGAUUTT-3’  5’-AAUCAUUGUAGACCUGAGGTT-3’ |
| siRNA negative control | 5’-UUCUCCGAACGUGUCACGUTT-3’  5’-ACGUGACACGUUCGGAGAATT-3’ |

**Supplementary Table S1. siRNA sequences used in this study.**

**Supplementary Table S2. PCR primer sequences used in this study.**

| Gene name | Primer ID | Primer Sequence (5'-3') |
| --- | --- | --- |
| LINC01279 | LINC01279-F | CAGGGTTGCCACAAACCTTC |
| LINC01279 | LINC01279-R | ATGCCAGTATGGTAATAGGTCCA |
| FAK | FAK-F | AGCCGCAGAGATCACCTTTCATTG |
| FAK | FAK-R | ATTGCTTGAGCCTGGGAGTTTGAG |
| ERK | ERK-F | ATGGTGTGCTCTGCTTATGATA |
| ERK | ERK-R | TCTTTCATTTGCTCGATGGTTG |
| SIN3A | SIN3A-F | CAGCTACGTCTCAAAGAACCTA |
| SIN3A | SIN3A-R | GCATGAATGGTGAACATCTCTC |
| GAPDH | GAPDH-F | GTCAAGGCTGAGAACGGGAA |
| GAPDH | GAPDH-R | AAATGAGCCCCAGCCTTCTC |
| U1 | U1-F | ACCTGGCAGGGGAGATACCA |
| U1 | U1-R | GAAAGCGCGAACGCAGTCC |

**Supplementary Table S3. Antibodies used in this study.**

| PARP (Cell Signaling Technology, 9532) |
| --- |
| p-AKT (Cell Signaling Technology, 4056) |
| p-ERK (Cell Signaling Technology, 4370) |
| ERK (Cell Signaling Technology, 9102) |
| GAPDH (Sigma, AB2302) |
| SIN3A (Cell Signaling Technology, 8056) |
| LC3B-II (abcam, ab192890) |
| FAK (Cell Signaling Technology, 3285) |
| p53 (abcam, ab32049) |
| p62 (Cell Signaling Technology, 5114) |
| p70 (Cell Signaling Technology, 2708) |
| Beclin1 (Cell Signaling Technology, 3495) |
| AMPK (Cell Signaling Technology, 5832) |
| p-AMPK (Cell Signaling Technology, 2535) |
| Ki-67 (Cell Signaling Technology, 9129) |
